# Crystal structure of 2-methyl-3-nitro­benzoic anhydride

**DOI:** 10.1107/S2056989015010531

**Published:** 2015-06-06

**Authors:** Rodolfo Moreno-Fuquen, Alexis Azcárate, Alan R. Kennedy

**Affiliations:** a Departamento de Química - Facultad de Ciencias Naturales y Exactas, Universidad del Valle, Apartado 25360, Santiago de Cali, Colombia; b WestCHEM, Department of Pure and Applied Chemistry, University of Strathclyde, 295 Cathedral Street, Glasgow G1 1XL, Scotland

**Keywords:** crystal structure, benzoic acid derivative, anhydrous compound, hydrogen bonding

## Abstract

The title mol­ecule, C_16_H_12_N_2_O_7_, lies on a twofold rotation axis which bis­ects the central O atom. The dihedral angle between two symmetry-related benzene rings is 48.54 (9)°. In the crystal, mol­ecules are linked by weak C—H⋯O hydrogen bonds which generate *C*(13) chains running parallel to [31-1].

## Related literature   

For related structures, see: Schmitt *et al.* (2011 ▸); Liu *et al.* (2009 ▸); Huelgas *et al.* (2006 ▸); Glówka *et al.* (1990 ▸). For hydrogen-bond details, see: Nardelli (1995 ▸).
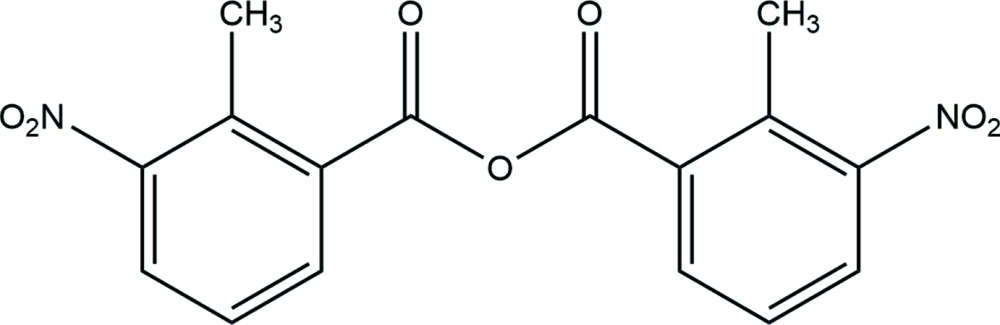


## Experimental   

### Crystal data  

C_16_H_12_N_2_O_7_
*M* *_r_* = 344.28
Monoclinic, 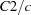

*a* = 10.6332 (5) Å
*b* = 11.6961 (4) Å
*c* = 12.7934 (6) Å
β = 111.930 (6)°
*V* = 1475.95 (12) Å^3^
*Z* = 4
Cu *K*α radiation
μ = 1.06 mm^−1^
*T* = 123 K
0.45 × 0.40 × 0.16 mm

### Data collection  

Oxford Diffraction Gemini S diffractometer
Absorption correction: multi-scan (*CrysAlis PRO*; Oxford Diffraction, 2010 ▸) *T*_min_ = 0.550, *T*_max_ = 1.000
5209 measured reflections
1466 independent reflections
1384 reflections with *I* > 2σ(*I*)
*R*_int_ = 0.101

### Refinement  

*R*[*F* ^2^ > 2σ(*F* ^2^)] = 0.059
*wR*(*F* ^2^) = 0.163
*S* = 1.08
1466 reflections
115 parameters
H-atom parameters constrained
Δρ_max_ = 0.40 e Å^−3^
Δρ_min_ = −0.33 e Å^−3^

### 

Data collection: *CrysAlis PRO* (Oxford Diffraction, 2010 ▸); cell refinement: *CrysAlis PRO*; data reduction: *CrysAlis PRO*; program(s) used to solve structure: *SIR92* (Altomare *et al.*, 1994 ▸); program(s) used to refine structure: *SHELXL2014* (Sheldrick, 2015 ▸); molecular graphics: *ORTEP-3 for Windows* (Farrugia, 2012 ▸) and *Mercury* (Macrae *et al.*, 2006 ▸); software used to prepare material for publication: *WinGX* (Farrugia, 2012 ▸).

## Supplementary Material

Crystal structure: contains datablock(s) I, global. DOI: 10.1107/S2056989015010531/lh5768sup1.cif

Structure factors: contains datablock(s) I. DOI: 10.1107/S2056989015010531/lh5768Isup2.hkl

Supporting information file. DOI: 10.1107/S2056989015010531/lh5768Isup3.cml

**Fig. 1.**
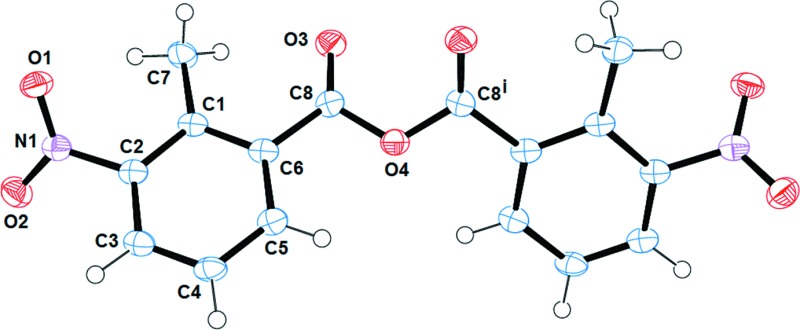
The molecular structure of (I) with displacement ellipsoids drawn at the 50% probability level. H atoms are shown as spheres of arbitrary radius (symmetry code: (i) -x, y, -z + 3/2).

Click here for additional data file.x y z . DOI: 10.1107/S2056989015010531/lh5768fig1.tif
The mol­ecular structure of (I) with displacement ellipsoids drawn at the 50% probability level. H atoms are shown as spheres of arbitrary radius (symmetry code: (i) −*x*, *y*, −*z* + 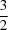).

**Fig. 2.**
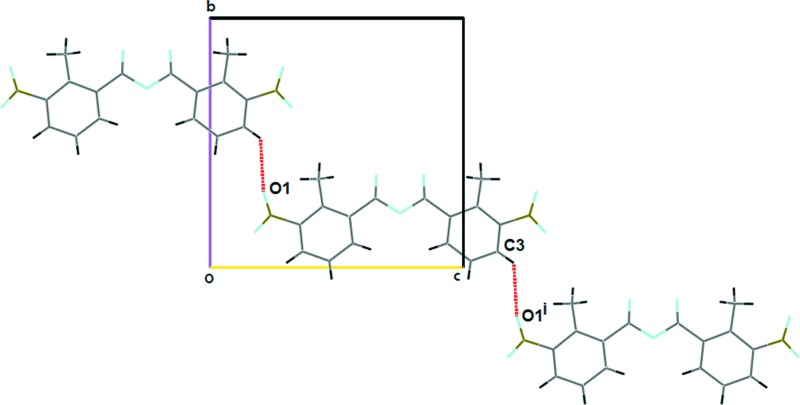
Part of the crystal structure of (I), showing the formation of a hydrogen-bonded C(13) chain parallel to [311] (symmetry code: (i) -x - 1/2 ,+y - 1/2, -z + 1/2).

Click here for additional data file.C x y z . DOI: 10.1107/S2056989015010531/lh5768fig2.tif
Part of the crystal structure of (I), showing the formation of a hydrogen-bonded *C*(13) chain parallel to [31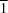] (symmetry code: (i) −*x* − 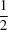, +*y* − 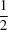, −*z* + 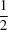).

CCDC reference: 1404417

Additional supporting information:  crystallographic information; 3D view; checkCIF report

## Figures and Tables

**Table 1:** Hydrogen-bond geometry (, )

*D*H*A*	*D*H	H*A*	*D* *A*	*D*H*A*
C3H3O1^i^	0.95	2.52	3.204(2)	129

Symmetry code: (i) 
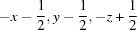
.

## supplementary crystallographic information

### S1. Comment

In the synthesis of phenyl-benzamides performed in our research group for quite some time, the untimely production of 2-methyl-3-nitrobenzoic anhydride (I) as a product of the reaction system was given. A small excess in moles of 2-methyl-3-nitrobenzoic acid in the presence of thionyl chloride in the reaction and the subsequent addition of the o-nitroaniline in dry acetonitrile, allowed the formation of two different types of crystals: the corresponding amide and the 2-methyl-3-nitrobenzoic anhydride. The excess addition of 2-methyl-3-nitrobenzoic acid possibly yield the benzyl halide formation which subsequently reacts with another molecule of acid, forming the anhydride system in dry condition. A number of anhydrous compounds, from benzoic acid derivatives are reported in the literature. Some with halogen substituents on the rings, encounter a widespread use as chelate ligands in coordination chemistry (Schmitt *et al.*, 2011). Similar compounds to (I) have been reported in the literature: N-phenylanthranilic anhydride (II) (Liu *et al.*, 2009), o-nitrobenzoic acid anhydride (III) (Huelgas *et al.*, 2006) and m-nitrobenzoic acid anhydride (IV) (Glówka *et al.*, 1990). The molecular structure of (I) is shown in Fig. 1. The central anhydride moiety C6-C8(═O3)-O4 shows a C8^i^-O4-C8-O3 torsion angle (symmetry code: (i) -x, y, -z+3/2) of 25.06 (14)°. The twofold rotation axis passes through atom O4. Bond lengths and bond angles in the molecule are in a good agreement with those found in the related compounds (II), (III) and (IV), with the exception of the C6—C8 bond length. The title structure exhibits strong elongation in the C6—C8 bond length [1.494 (2) Å] if compared to the similar bond length presented in (III) [1.402 (2) Å]. In the crystal structure (Fig. 2), molecules are linked by weak C—H···O hydrogen bonds (see Table 1, Nardelli, 1995). The C3—H3 group in the molecule at (x,y,z) acts as hydrogen bond donor to O1 atom of the nitro group in the molecule at (-x-1/2,+y-1/2,-z+1/2). These interactions generate C(13) chains of molecules parallel to [3 11].

### S2. Experimental

A mass of 0.380 g (1.104 mmol) of 2-methyl-3-nitrobenzoic acid was refluxed with 2 ml of thionyl chloride for one hour. Then 0.125 g (0.906 mmol) of 2-nitroaniline was added and dissolved in 10 ml of dry acetonitrile and it was placed under reflux and constant stirring for 3 hours. Subsequently, the final solvent was slowly evaporated to obtain colorless blocks of the title compound [m.p. 428 (1)K], and other yellow crystals of the amide show a melting point of 399 (1)K.

### S3. Refinement

All H-atoms were located in difference Fourier maps and were positioned geometrically [C—H = 0.95 Å for aromatic, C—H= 0.98 Å for methyl] and were refined using a riding-model approximation with *U*_iso_(H) constrained to 1.2 times *U*_eq_ of the respective parent atom or 1.2 times *U*_eq_(C_methyl_).

### Crystal data

**Table:**

C_16_H_12_N_2_O_7_	*D*_x_ = 1.549 Mg m^−^^3^
*M**_r_* = 344.28	Melting point: 428(1) K
Monoclinic, *C*2/*c*	Cu *K*α radiation, λ = 1.54180 Å
*a* = 10.6332 (5) Å	Cell parameters from 5209 reflections
*b* = 11.6961 (4) Å	θ = 5.9–73.2°
*c* = 12.7934 (6) Å	µ = 1.06 mm^−^^1^
β = 111.930 (6)°	*T* = 123 K
*V* = 1475.95 (12) Å^3^	Block, colourless
*Z* = 4	0.45 × 0.40 × 0.16 mm
*F*(000) = 712	

### Data collection

**Table:**

Oxford Diffraction Gemini S diffractometer	1466 independent reflections
Radiation source: fine-focus sealed tube	1384 reflections with *I* > 2σ(*I*)
Graphite monochromator	*R*_int_ = 0.101
ω scans	θ_max_ = 73.2°, θ_min_ = 5.9°
Absorption correction: multi-scan (*CrysAlis PRO*; Oxford Diffraction, 2010)	*h* = −12→13
*T*_min_ = 0.550, *T*_max_ = 1.000	*k* = −14→14
5209 measured reflections	*l* = −14→15

### Refinement

**Table:**

Refinement on *F*^2^	Primary atom site location: structure-invariant direct methods
Least-squares matrix: full	Secondary atom site location: difference Fourier map
*R*[*F*^2^ > 2σ(*F*^2^)] = 0.059	Hydrogen site location: inferred from neighbouring sites
*wR*(*F*^2^) = 0.163	H-atom parameters constrained
*S* = 1.08	*w* = 1/[σ^2^(*F*_o_^2^) + (0.0933*P*)^2^ + 1.5216*P*] where *P* = (*F*_o_^2^ + 2*F*_c_^2^)/3
1466 reflections	(Δ/σ)_max_ < 0.001
115 parameters	Δρ_max_ = 0.40 e Å^−^^3^
0 restraints	Δρ_min_ = −0.33 e Å^−^^3^

### Special details

**Table:**

Geometry. All esds (except the esd in the dihedral angle between two l.s. planes) are estimated using the full covariance matrix. The cell esds are taken into account individually in the estimation of esds in distances, angles and torsion angles; correlations between esds in cell parameters are only used when they are defined by crystal symmetry. An approximate (isotropic) treatment of cell esds is used for estimating esds involving l.s. planes.
Refinement. Refinement of *F*^2^ against ALL reflections. The weighted *R*-factor *wR* and goodness of fit *S* are based on *F*^2^, conventional *R*-factors *R* are based on *F*, with *F* set to zero for negative *F*^2^. The threshold expression of *F*^2^ > σ(*F*^2^) is used only for calculating *R*-factors(gt) *etc*. and is not relevant to the choice of reflections for refinement. *R*-factors based on *F*^2^ are statistically about twice as large as those based on *F*, and *R*- factors based on ALL data will be even larger.

### Fractional atomic coordinates and isotropic or equivalent isotropic displacement parameters (Å^2^)

**Table:**

	*x*	*y*	*z*	*U*_iso_*/*U*_eq_	
O1	−0.22053 (15)	0.30472 (11)	0.21092 (12)	0.0323 (4)	
O2	−0.15352 (15)	0.13946 (12)	0.17543 (12)	0.0348 (4)	
O3	0.03327 (14)	0.37124 (12)	0.65687 (11)	0.0303 (4)	
O4	0.0000	0.21684 (16)	0.7500	0.0270 (5)	
N1	−0.17462 (15)	0.20860 (13)	0.23928 (13)	0.0247 (4)	
C1	−0.08126 (16)	0.24457 (15)	0.44518 (15)	0.0214 (4)	
C2	−0.14895 (17)	0.17107 (15)	0.35513 (15)	0.0223 (4)	
C3	−0.19730 (18)	0.06382 (15)	0.36540 (16)	0.0254 (4)	
H3	−0.2393	0.0172	0.3009	0.031*	
C4	−0.18356 (18)	0.02561 (16)	0.47094 (16)	0.0275 (4)	
H4	−0.2150	−0.0482	0.4804	0.033*	
C5	−0.12307 (18)	0.09647 (16)	0.56351 (15)	0.0252 (4)	
H5	−0.1164	0.0716	0.6361	0.030*	
C6	−0.07208 (17)	0.20339 (14)	0.55154 (15)	0.0219 (4)	
C7	−0.0176 (2)	0.35437 (16)	0.42891 (16)	0.0284 (5)	
H7A	−0.0092	0.3536	0.3552	0.043*	
H7B	0.0724	0.3623	0.4883	0.043*	
H7C	−0.0747	0.4189	0.4326	0.043*	
C8	−0.00896 (18)	0.27684 (16)	0.65334 (15)	0.0231 (4)	

### Atomic displacement parameters (Å^2^)

**Table:**

	*U*^11^	*U*^22^	*U*^33^	*U*^12^	*U*^13^	*U*^23^
O1	0.0356 (8)	0.0225 (7)	0.0357 (8)	0.0027 (6)	0.0095 (6)	0.0041 (5)
O2	0.0382 (9)	0.0322 (8)	0.0336 (8)	0.0025 (6)	0.0130 (6)	−0.0055 (5)
O3	0.0288 (7)	0.0253 (7)	0.0315 (7)	−0.0084 (5)	0.0051 (6)	−0.0010 (5)
O4	0.0288 (10)	0.0222 (9)	0.0279 (9)	0.000	0.0082 (8)	0.000
N1	0.0188 (8)	0.0233 (8)	0.0299 (8)	−0.0014 (6)	0.0067 (6)	−0.0012 (6)
C1	0.0126 (7)	0.0194 (8)	0.0307 (9)	0.0006 (6)	0.0062 (6)	−0.0009 (7)
C2	0.0150 (8)	0.0207 (9)	0.0293 (9)	0.0012 (6)	0.0061 (7)	0.0002 (7)
C3	0.0170 (8)	0.0218 (9)	0.0327 (9)	−0.0007 (7)	0.0038 (7)	−0.0026 (7)
C4	0.0216 (8)	0.0199 (8)	0.0362 (10)	−0.0046 (7)	0.0052 (7)	0.0012 (7)
C5	0.0178 (8)	0.0247 (9)	0.0301 (9)	−0.0017 (7)	0.0054 (7)	0.0023 (7)
C6	0.0121 (7)	0.0202 (8)	0.0310 (10)	0.0002 (6)	0.0054 (7)	−0.0008 (6)
C7	0.0292 (10)	0.0253 (9)	0.0312 (9)	−0.0093 (7)	0.0118 (8)	−0.0030 (7)
C8	0.0146 (8)	0.0247 (9)	0.0272 (9)	0.0002 (7)	0.0047 (6)	0.0018 (7)

### Geometric parameters (Å, º)

**Table:**

O1—N1	1.225 (2)	C3—C4	1.377 (3)
O2—N1	1.228 (2)	C3—H3	0.9500
O3—C8	1.186 (2)	C4—C5	1.391 (3)
O4—C8	1.3939 (19)	C4—H4	0.9500
O4—C8^i^	1.3939 (19)	C5—C6	1.394 (2)
N1—C2	1.470 (2)	C5—H5	0.9500
C1—C2	1.402 (2)	C6—C8	1.494 (2)
C1—C6	1.412 (3)	C7—H7A	0.9800
C1—C7	1.502 (2)	C7—H7B	0.9800
C2—C3	1.380 (2)	C7—H7C	0.9800
			
C8—O4—C8^i^	119.5 (2)	C4—C5—C6	121.08 (17)
O1—N1—O2	123.96 (16)	C4—C5—H5	119.5
O1—N1—C2	118.41 (15)	C6—C5—H5	119.5
O2—N1—C2	117.56 (15)	C5—C6—C1	121.47 (16)
C2—C1—C6	114.35 (16)	C5—C6—C8	119.12 (16)
C2—C1—C7	122.01 (16)	C1—C6—C8	119.39 (15)
C6—C1—C7	123.55 (15)	C1—C7—H7A	109.5
C3—C2—C1	125.05 (17)	C1—C7—H7B	109.5
C3—C2—N1	115.58 (15)	H7A—C7—H7B	109.5
C1—C2—N1	119.37 (15)	C1—C7—H7C	109.5
C4—C3—C2	118.77 (16)	H7A—C7—H7C	109.5
C4—C3—H3	120.6	H7B—C7—H7C	109.5
C2—C3—H3	120.6	O3—C8—O4	122.37 (16)
C3—C4—C5	119.17 (17)	O3—C8—C6	127.50 (16)
C3—C4—H4	120.4	O4—C8—C6	110.09 (15)
C5—C4—H4	120.4		
			
C6—C1—C2—C3	−3.8 (3)	C4—C5—C6—C1	0.8 (3)
C7—C1—C2—C3	172.93 (16)	C4—C5—C6—C8	179.52 (16)
C6—C1—C2—N1	175.09 (14)	C2—C1—C6—C5	2.1 (2)
C7—C1—C2—N1	−8.2 (2)	C7—C1—C6—C5	−174.58 (16)
O1—N1—C2—C3	132.42 (17)	C2—C1—C6—C8	−176.64 (14)
O2—N1—C2—C3	−44.8 (2)	C7—C1—C6—C8	6.7 (3)
O1—N1—C2—C1	−46.6 (2)	C8^i^—O4—C8—O3	25.06 (14)
O2—N1—C2—C1	136.26 (17)	C8^i^—O4—C8—C6	−157.20 (15)
C1—C2—C3—C4	2.4 (3)	C5—C6—C8—O3	−176.29 (18)
N1—C2—C3—C4	−176.46 (16)	C1—C6—C8—O3	2.5 (3)
C2—C3—C4—C5	0.8 (3)	C5—C6—C8—O4	6.1 (2)
C3—C4—C5—C6	−2.3 (3)	C1—C6—C8—O4	−175.15 (14)

Symmetry code: (i) −*x*, *y*, −*z*+3/2.

### Hydrogen-bond geometry (Å, º)

**Table:**

*D*—H···*A*	*D*—H	H···*A*	*D*···*A*	*D*—H···*A*
C3—H3···O1^ii^	0.95	2.52	3.204 (2)	129

Symmetry code: (ii) −*x*−1/2, *y*−1/2, −*z*+1/2.
