# Supplementary figures and images for: Crystal structure of 2-methyl-3-nitro­benzoic anhydride
Source: Acta Crystallogr E Crystallogr Commun. 2015 Jun 6;71(Pt 7):o451. doi: 10.1107/S2056989015010531 (PMC4518930; doi:10.1107/S2056989015010531)

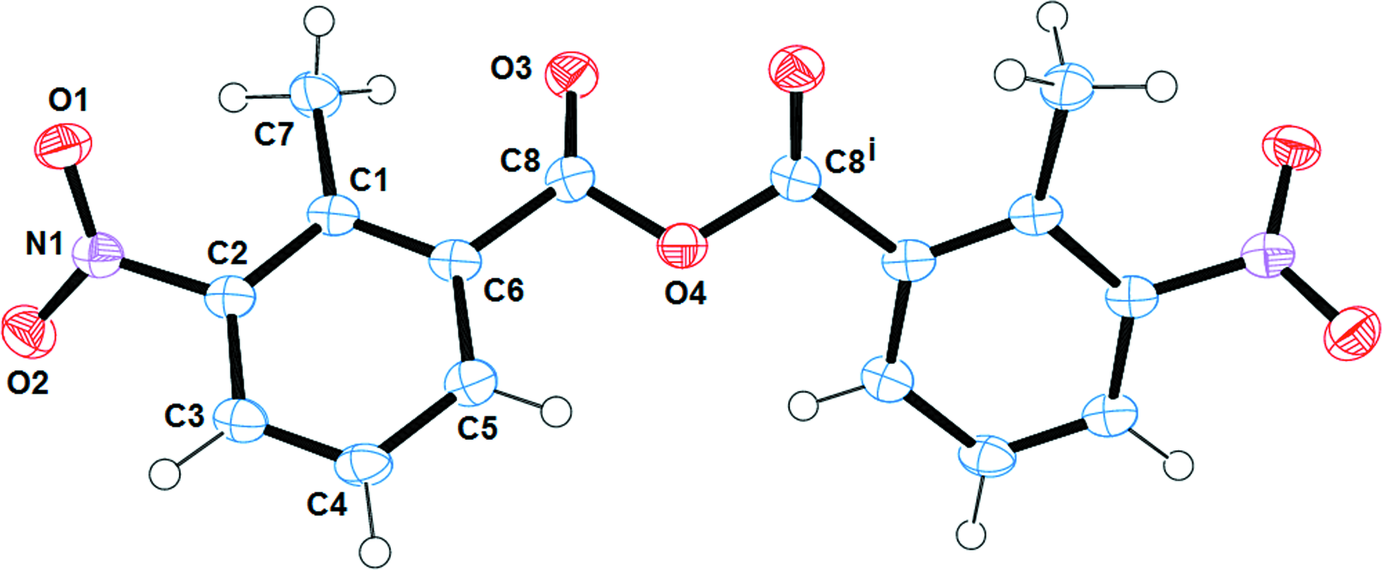

Supplement: Supplementary file 4 [file e-71-0o451-fig1.tif]

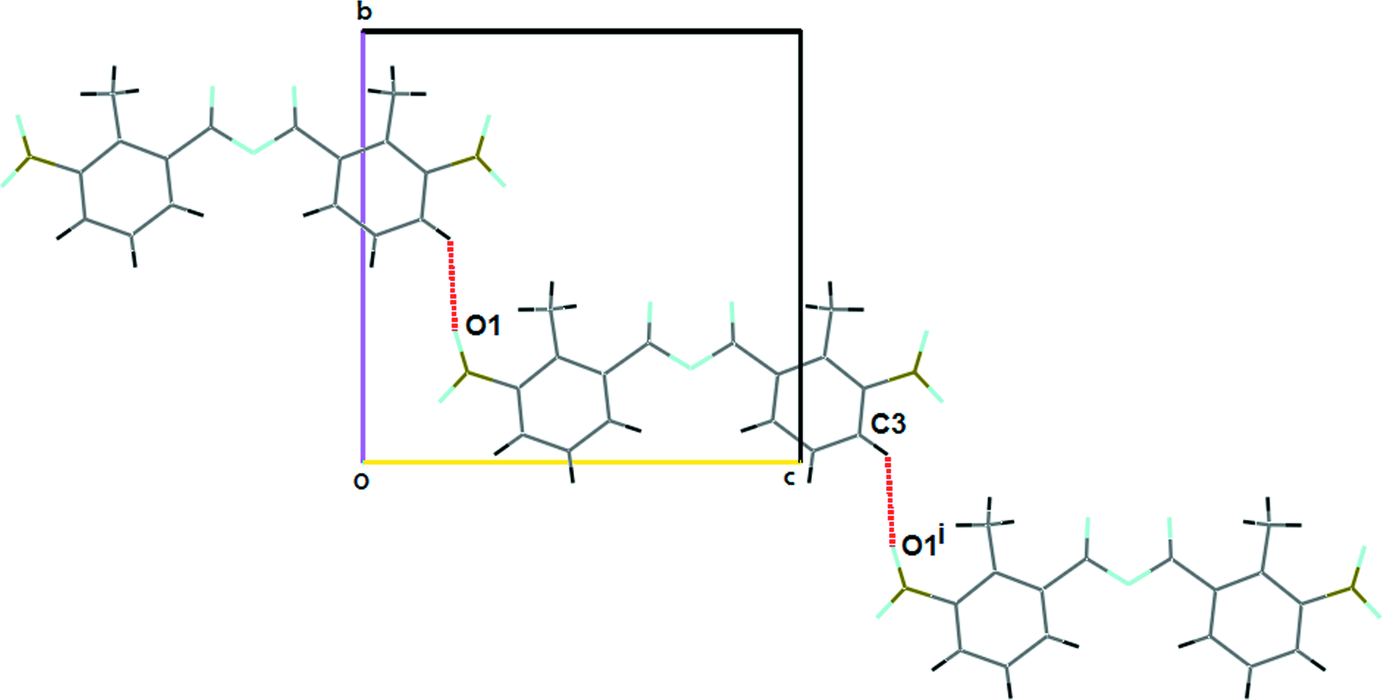

Supplement: Supplementary file 5 [file e-71-0o451-fig2.tif]
